# Gene expression profile of cervical tissue compared to exfoliated cells: Impact on biomarker discovery

**DOI:** 10.1186/1471-2164-6-64

**Published:** 2005-05-05

**Authors:** Martin Steinau, Daisy R Lee, Mangalathu S Rajeevan, Suzanne D Vernon, Mack T Ruffin, Elizabeth R Unger

**Affiliations:** 1 Centers for Disease Control and Prevention, Atlanta, GA, USA; 2 University of Michigan, Ann Arbor, MI, USA

## Abstract

**Background:**

Exfoliated cervical cells are used in cytology-based cancer screening and may also be a source for molecular biomarkers indicative of neoplastic changes in the underlying tissue. However, because of keratinization and terminal differentiation it is not clear that these cells have an mRNA profile representative of cervical tissue, and that the profile can distinguish the lesions targeted for early detection.

**Results:**

We used whole genome microarrays (25,353 unique genes) to compare the transcription profiles from seven samples of normal exfoliated cells and one cervical tissue. We detected 10,158 genes in exfoliated cells, 14,544 in the tissue and 7320 genes in both samples. For both sample types the genes grouped into the same major gene ontology (GO) categories in the same order, with exfoliated cells, having on average 20% fewer genes in each category. We also compared microarray results of samples from women with cervical intraepithelial neoplasia grade 3 (CIN3, n = 15) to those from age and race matched women without significant abnormalities (CIN1, CIN0; n = 15). We used three microarray-adapted statistical packages to identify differential gene expression. The six genes identified in common were two to four fold upregulated in CIN3 samples. One of these genes, the ubiquitin-conjugating enzyme E2 variant 1, participates in the degradation of p53 through interaction with the oncogenic HPV E6 protein.

**Conclusion:**

The findings encourage further exploration of gene expression using exfoliated cells to identify and validate applicable biomarkers. We conclude that the gene expression profile of exfoliated cervical cells partially represents that of tissue and is complex enough to provide potential differentiation between disease and non-disease.

## Background

Early cancer detection requires noninvasive sampling is for general screening populations. Exfoliated cervical cells have been used for cytologic screening of cervical cancer. These accessible cells could also be ideal for molecular screening based on gene expression if their mRNA can be isolated and is representative of the expression profile of the underlying tissue. We have previously shown that satisfactory RNA can be isolated from pap smear material in amounts sufficient for microarray analysis [1]. However since exfoliated cells are keratinized and terminally differentiated, it remains to be demonstrated that they have an active mRNA profile that adequately represents the molecular signature of cervical tissue. While one group reported success using exfoliated oral cells as a source of gene expression biomarkers [2], others have not obtained satisfactory results [3].

This study addresses the representation of gene expression profiles in exfoliated cells and full thickness epithelium. To further explore the possibility of using exfoliated cells for molecular screening, we compared the gene expression in samples with cervical intraepithelial neoplasia grade 3 (CIN3) to those without or only low grade lesions (CIN0, CIN1).

## Results

### Gene expression profiles in cervical exfoliated cells and tissue

**Figure 1:**
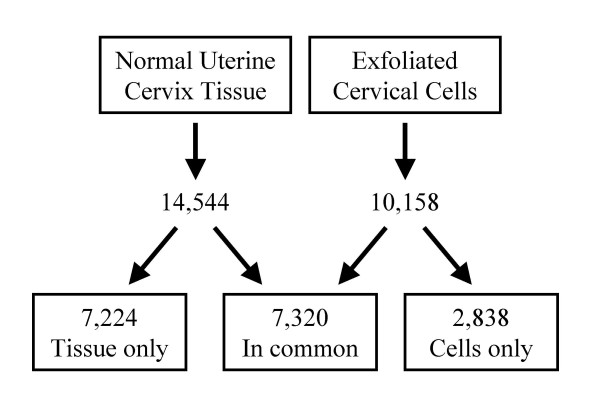
Number of genes expressed above cutoff in cervical tissue and exfoliated cells.

**Figure 2:**
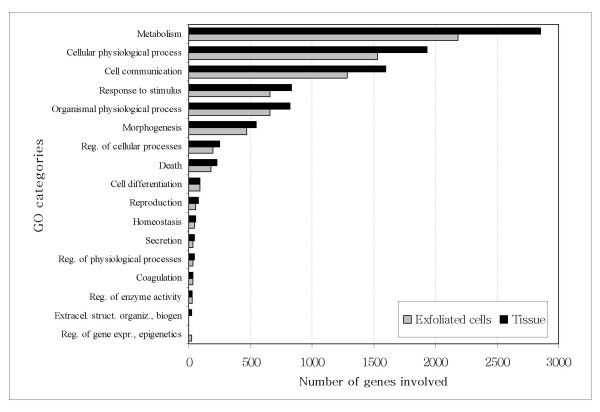
**GOs of biological processes in cervical exfoliated cells and full thickness epithelium. **While expressed genes represent major categories in the same order, a proportion of each (4.4 to 27.3 %) is not present in exfoliated cells.

The MWG A, B and C human 30 k arrays include probes evaluating 25,353 different genes of the transcribed human genome. Of these genes, 14,544 (57%) were detected in uterine cervix tissue and 10,158 (40%) in exfoliated cells (Figure 1). Of those detected in tissue, 7,320 (50.3%) were also detected in exfoliated cells. Genes detected in each sample type grouped into the same major GO categories in the same order of abundance (Figure 2), although the numbers in each category were on average 20% less in exfoliated cells (range 4.4 – 27.3%). The exception is the GO category, regulation of gene expression, epigenetics, in which the few genes that were detected were only found in exfoliated cells.

EASE analysis of the 7,224 genes detected only in cervical tissue found the biologic themes of structural cell components, ribosomal function and structure, ion transport and regulation to be enriched relative to their representation on the arrays (Table 1). Biologic themes enriched in genes detected only in exfoliated cells were mainly differentiation processes like neurogenesis, morphogenesis, oncogenesis, organogenesis and development (Table 2).

**Table 1:** Biological themes enriched in genes detected only in tissue

	Gene Category	# of Genes	EASE score^†^
Molecular Function	Structural molecule activity	228	2.27E-07
	Structural constituent of ribosome	78	4.21E-05
	Extracellular matrix structural constituent	38	4.12E-04
	Monovalent cation \:proton antiporter activity	7	3.90E-03
	Sodium \:hydrogen antiporter activity	7	3.90E-03
	Transcriptional repressor activity	18	1.31E-02
	Voltage-gated sodium channel activity	9	1.58E-02
	Cation \:cation antiporter activity	8	2.24E-02

Cellular Component	Large ribosomal subunit	23	1.06E-03
	Ribosome	90	2.20E-03
	Cytosolic ribosome	24	6.14E-03
	Cytosolic large ribosome subunit	16	9.87E-03
	Cellular component unknown	160	1.22E-02
	Collagen	15	1.36E-02
	Ribonucleoprotein complex	127	1.52E-02

	Biological process unknown	252	7.91E-04

^†^Cut-off <0.025

**Table 2:** Biological themes enriched in genes detected only in exfoliated cells

	Gene Category	# of Genes	EASE score^†^
Biological process	Neurogenesis	62	6.46E-04
	Development	205	1.38E-03
	Behavior	18	6.30E-03
	Morphogenesis	122	1.04E-02
	Regulation of cell adhesion	7	1.10E-02
	Oncogenesis	14	1.38E-02
	Cellular process	614	1.82E-02
	Organogenesis	108	1.82E-02

*	Chromatin remodeling complex	10	6.73E-03
	Cell fraction	91	2.44E-02

**	Protein tyrosine/serine/threonine phosphatase activity	91	3.23E-03

^†^Cut-off <0.025; *Cellular Component; **Molecular Function

### Gene expression comparison of CIN3 samples with CIN0 and CIN1 controls

**Figure 3:**
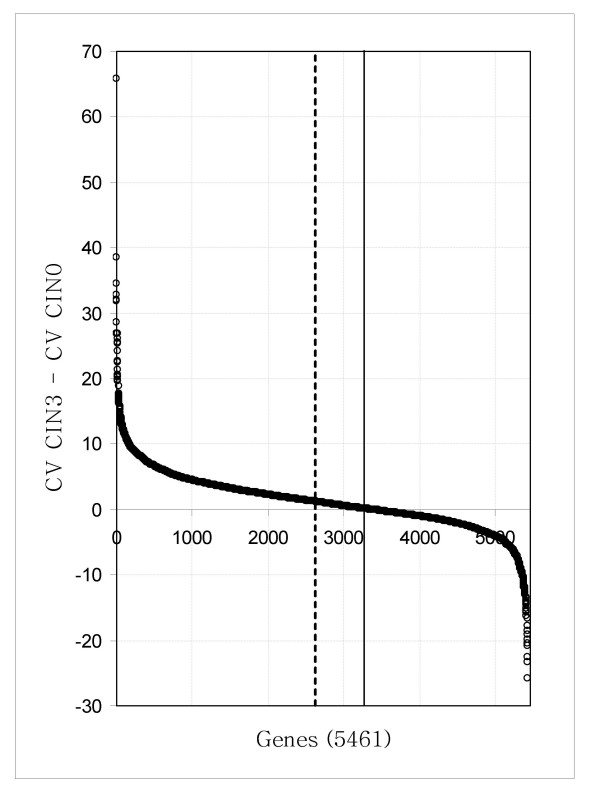
**Plot of CV differences between CIN3 and CIN0 samples. **CVs were calculated from normalized, log_2 _transformed sARM values for each gene. If variance in gene expression was random in both groups, approximately half the genes would be more variable in CIN 3 (positive) and half in CIN 0 (negative). The vertical dashed line divides the total number of genes in half (at gene 2730), the solid line marks where were the CVs are equal (at gene 3364). The increased number of genes with greater CV in the CIN 3 group indicates greater heterogeneity of expression profiles in this disease group compared with the control (CIN 0) group.

Of the 5461 genes on the A arrays that were included in this analysis, the average expression of 95 were at least 2-fold greater whereas 10 were at least 2-fold lower in CIN3 compared to CIN 0/CIN 1. Based on CV, the CIN 3 class showed more heterogeneity than the CIN 0 class (Figure 3) or the combined CIN 0/CIN 1 controls (data not shown).

The univariate parametric p-values of the 20 most significant genes that discriminate between the CIN3 and the CIN 0/CIN 1 groups selected in the BRB Array Tools two-sample T-test ranged between 0.00077 – 0.0067316. None had a false discovery rate (FDR) of less than 10%. The GO class comparison yielded 11 categories significant at the nominal 0.005 level of permutation tests. These were helicase activity, replication fork, DNA-directed DNA polymerase activity, regulation of viral life cycle, peroxidase activity, DNA replication and endoplasmatic reticulum membrane, or a closely related subcategory (Table 3).

**Table 3:** GO categories with higher than expected numbers of genes differentially regulated in CIN3(p < 0.005).

**GO category**	**GO description**	**LS Permut. p-value**	**KS Permut. p-value**
0004376	Helicase activity	0.00129	0.00718
0001724	RNA helicase activity	0.00196	0.01317
0030894	Replisome	0.00811	1e-04
0005657	Replication fork	0.01098	7e-04
0003887	DNA-directed DNA polymerase activity	0.01822	0.00253
0050792	Regulation of viral life cycle	0.03079	0.00364
0004601	Peroxidase activity	0.03864	0.00382
0016684	Oxidoreductase activity	0.03864	0.00382
0006260	DNA replication	0.06296	0.00403
0006270	DNA replication initiation	0.07700	0.00066
0004789	Endoplasmic reticulum membrane	0.09838	0.00409

SAM yielded a trimmed list of 14 genes with a median FDR of 35.7. Nine of these genes were also in the top list generated with BRB Tools. Among the first 21 genes that Focus identified as agreeing best with the hypothesized pattern change, 12 were seen in BRB Array Tools top list and 9 by SAM. Table 4 shows the six genes identified by all three methods. All were upregulated in CIN3 by an average fold change of 2.3 (2.0 – 3.3).

**Table 4:** Genes with statistically significant upregulated expression in CIN3. Six genes were indicated by all three analysis methods (BRB Array-Tools, SAM, Focus). Fold changes are shown as calculated by SAM.

**GenBank ID**	**Gene Name**	**Fold Change**
NM_021988	ubiquitin-conjugating enzyme E2 variant 1	2.0
NM_018307	ras homolog gene family, member T1	2.1
NM_024719	growth hormone regulated TBC protein 1	2.3
NM_007083	nudix type motif 6	2.1
NM_022841	hypothetical protein FLJ12994	3.3
NM_016175	truncated calcium binding protein	2.0

## Discussion

Exfoliated cells from normal squamous epithelium are derived from the terminally differentiated superficial layers and may have a more restricted representation of the underlying tissue than those derived from a neoplastic epithelium where differentiation is reduced or lost completely. Therefore results from the normal samples should provide a conservative estimate of the degree of similarities between cells and tissue. Grouping the detected genes by broad ontology categories, the cervical tissue and cervical exfoliated samples showed a similar distribution, however exfoliated cells had fewer numbers of genes in each category. The genes in common between tissue and exfoliated cell profiles represented 50% of the total genes detected in tissue and 72% of the total detected in the cells. Therefore, while much of the tissue gene profile is included in the exfoliated cells, this representation is only partial.

The 7224 genes that were not detected in exfoliated cells could be expected to be involved in proliferation of the basal epithelium. Even though the GOs of these genes did not indicate a direct involvement of these genes in cell division they did include basic components of cell structure and function. Interestingly, 6 of the 15 most significant GOs were related to ribosome activity implying that the ribosomal complex is not renewed after initial establishment in the basal cells.

It is interesting to note that 2,838 genes found in the exfoliated samples were not detected in the tissue. One explanation may be the presence of inflammatory or other cells such as endometrium that are not present in tissue. With keratinocyte differentiation the profile becomes more specialized as some genes are down regulated [4]. Therefore, another explanation may be that the restricted RNA profile allows genes below the cutoff in tissue to exceed the threshold for detection in exfoliated cells. This is supported by the fact that average signal intensity (sARM) of these genes was only one third of these expressed in both specimens. The observation that the GOs of these genes were relate to advanced differentiation processes favors the latter explanation.

Since we used only one cervical tissue sample we undoubtedly underestimated the true biologic variability introduced by age, hormonal status and other environmental factors. In addition, requiring detection in 6 of 7 exfoliated samples limits the transcriptome to those genes that could reliably and reproducibly be detected by microarrays. Additional study, including cervical tissues and matched exfoliated samples from a spectrum of disease states would be required to fully define the extent to which tissue and exfoliated cell profiles overlap, nonetheless we conclude that exfoliated cells are worthy of further investigation as a source of molecular markers for screening.

To begin biomarker discovery we conducted a pilot microarray study of exfoliated cells from women with CIN3 to those with no disease (CIN0) or CIN 1 to determine if differential gene expression could be identified and evaluate the degree of variation within disease groups to determine the number of samples that would be required to stabilize selection of differentially expressed genes. Not surprisingly, the gene expression profiles of the two groups were very similar. Based on cytology, less than 10% of the sampled cells are neoplastic, so the dilution effect on abnormal transcript profiles could be considerable. A "field effect", that is extension of molecular changes to an area larger than the histologically identifiable lesion, has been demonstrated in other cancers including head and neck [5], colon [6] and bladder [7]. There is some evidence that this may occur in the cervix [8], but the extent to which this occurs is not clear.

Using three different statistical approaches to identify differentially expressed genes resulted in lists of candidate genes with little overlap and relatively high estimates of false discovery rates. These inconsistencies reflect the relatively small differences between disease classes compared to within-class variation. We intentionally represented a wide range of age and race in this pilot so as not to over simplify the within-class variation thereby maximizing the specificity of the identified candidate biomarkers. Interestingly, despite matching age and race between the disease groups, the within-class variation was greater for CIN 3 than for no disease or combined CIN 0 and CIN 1. This suggests that cytologically identical CIN3 lesions may represent different molecular pathways to oncogenesis.

There were 6 genes that were identified by all three analysis methods. Given the central role of HPV in cervical carcinogenesis it is interesting that one of these, the ubiquitin-conjugating enzyme E2 variant 1, participates in the degradation of p53 through interaction with HPV E6 protein [9]. Similarly, it is interesting to note an over-representation of genes in the GO "viral life cycle" in CIN3. While none of the others have been previously implicated in cervical carcinogenesis this is not too surprising, as their role may be restricted to premalignant lesions or to host response.

## Conclusion

The primary goal of this pilot study was to explore the possibility of using exfoliated cells for genomic biomarker discovery. We conclude that RNA from these cells can indeed be applied to genomic studies. Exfoliated cells display an expression profile that reflects the tissue albeit with limited complexity. In addition, the characteristic expression differences between CIN 3 and control samples (CIN 1 and no disease) are small and future studies need to be designed to address these factors.

## Methods

### Study population

The study population consisted of women enrolled into an ongoing study of cervical neoplasia in high-risk urban women [10]. Participants were recruited from non-pregnant, HIV-negative women, aged 18–69 years, attending colposcopy clinics at urban public hospitals in Atlanta, Georgia and Detroit, Michigan. Specimens used in this study were from participants enrolled between December 2000 and December 2002. Cervical disease status was determined based on the summary results of cytology, colposcopy and biopsy examination. We selected 15 women with high grade lesions (CIN3) as cases and age (± 4 years) and race matched women without or with only low grade lesions as controls (7 CIN0 and 8 CIN1).

### Sample collection and RNA extraction

After visualization of the cervix, ecto- and endocervical cells were collected using a CytoBroom (Cytyc Corporation, Malborough, MA) and dislodged into PreservCyt collection media (Cytyc Corporation). If a cytology diagnosis was required, the collection device was used to prepare a conventional Pap smear and then placed into the PreservCyt collection media. Samples were transported to the laboratory at ambient temperature and stored at 4°C until processed. Within two weeks of sample collection, total nucleic acids (TNA) were extracted from 14 ml of each 20 ml PreservCyt sample using modifications of the MasterPure Complete DNA and RNA Purification kit (Epicentre, Madison, WI) as previously described (Habis et al 2004). The TNA extract was resuspended in 50 μL TE buffer with 50 units of RNasin (Promega Corporation, Madison, WI) and stored at -70°C until use. Total RNA derived from normal uterine cervix tissue (age 48, unknown ethnicity) was purchased (Stratagene^®^, La Jolla, CA). Quality of all samples was visually evaluated by gel electrophoresis and quantitation was assessed by densitometric measurement (FluorChem^® ^Digital Imaging System, Alpha Innotech, Inc., San Leandro, CA) of the ribosomal bands, with comparison to a standard 28S and 18S control marker.

### Microarray assays

We used MWG Human 30 k Arrays (A/ B/ C) (MWG Biotech, Ebersberg, Germany). Each array was hybridized with cDNA prepared from 500 ng total RNA. Conditions for labeling and hybridization were as described elsewhere [11]. Briefly, samples were pretreated with DNase I and cDNA was prepared and labeled with biotin-11-dUTP (Enzo, Farmingdale, NY) using SuperScript™ First-Strand Synthesis System for RT-PCR (Invitrogen, Carlsbad, CA) with oligo dT and random primers. The automated Discovery™ System (Ventana Medical Systems, Tucson, AZ) was used to hybridize slides for 8 hours at 42°C, and detect hybridization with anti-biotin Gold Resonance Light Scattering (RLS) Particles (Invitrogen). Slides were scanned with the GSD-501™ RLS scanner (Invitrogen) and 16-Bit Tiff images were subsequently quantified with Array Vision™ Software 8.0 (Imaging Research, St Catherines, ON, Canada). We used sARM values (artifact removed density minus the background density) of each feature for statistical analysis and a signal to noise ratio (S/N = sARM, divided by the SD of the background density) of 1.5 as the cut-off for detection of gene expression.

### Comparison of gene expression in cervical exfoliated cells and uterine cervix tissue

We used the results of the 7 samples of exfoliated cells from women with no abnormalities (CIN0) to characterize the profile of exfoliated cells and of the uterine cervix tissue RNA assayed in duplicate to characterize the tissue profile. A gene was included in the profile if detected in >85% of the exfoliated samples (6 of 7) or in both replicates of the tissue sample. We used the web based database for annotation, visualization and integrated discovery (DAVID) [12] for functional annotation and ontology of the detected genes, and the expression analysis systematic explorer (EASE) for identification of enriched biological themes within the gene lists as reflected by an EASE score of < 0.025 [13].

### Differential gene expression in CIN0/CIN1 and CIN3 samples

Expression data were derived from the results of all 30 exfoliated samples hybridized to MWG A arrays. Features were restricted to those with a S/N above 1.5 detected in at least 12 of the 15 samples (80%) of either class (CIN0/CIN1 or CIN3). 5461 genes that passed this filter were subjected to further statistical analysis.

We calculated the mean and coefficient of variation (CV) of the log_2 _transformed median centered sARM for each gene within the CIN 3, CIN 0 and CIN 1/CIN 0 groups. We used the CV in expression of each gene as a measure of homogeneity. For each gene we calculated the difference between the CV of the CIN 3 group minus that of the other groups and plotted these values in descending order to visualize discrepancies in the variation of genes expression between the classes.

To identify expression differences between the two groups we used the following three different microarray-adapted statistical software packages:

(1) BRB Array Tools 3.2 : Log_2 _transformed sARM values were normalized over the median of each array and subjected to a two-sample T-test with a random variance model and 1000 permutations. We considered the top 20 genes with the lowest univariate parametric p-values as differentially expressed. Multivariate permutation tests were applied to estimate the proportion of false discoveries in the discovery list. The indicated genes were assigned to gene ontology (GO) categories. Additionally, all GO categories that included at least 5 genes represented on the microarray were analyzed to identify biological themes overrepresented in genes differentially expressed. A GO category was selected if its corresponding LS or KS permutation p-value was below the threshold of 0.005 [14].

(2) Significant analysis of microarrays (SAM) 1.21 : sARM values were log_2 _transformed and centered to the median of each array. Normalized data were analyzed in an unpaired, two-class model with gene specific t-tests and 200 permutations to estimate false discovery rate (FDR) from multiple testing. Genes were scored based on change in expression relative to the standard deviation of the repeated measurements in order to identify those with differential expression [15].

(3) Focus 5.1 : Raw sARM data were normalized to a modified Z-transformation and tested for the hypothesis, gene expression in CIN3 samples is upregulated over controls. The applied contrast coefficient of 1.0 scored genes directly according to the average intensity difference between the two classes [16]. Genes agreeing best with the hypothesized pattern change were trimmed to a list of 20 with the highest interest scores and at least a 2-fold change.

## Authors' contributions

MS performed the microarray experiments, statistical analysis and bioinformatics. DRL organized sample collection and processing. MSR, SDV and MTR participated in aspects of the study design and contributed to the discussion and interpretations of the results. ERU initiated the project participated in its design and coordination and helped drafting the manuscript.

All authors read and approved the final manuscript.
